# Determining the Impact of the COVID-19 Pandemic on Availability, Use, and Readiness of Family Planning and Contraceptive Services at Selected Primary Health Care Facilities in Africa and Asia: Protocol for a Mixed Methods Study

**DOI:** 10.2196/43329

**Published:** 2023-05-10

**Authors:** Rita Kabra, Beena Joshi, Ester Elisaria, Tanimola Makanjuola Akande, Komal Preet Allagh, Adesola Olumide, Deepti Tandon, Ranjan Kumar Prusty, Mary Ramesh, Donat Shamba, James Kiarie

**Affiliations:** 1 UNDP-UNFPA-UNICEF-WHO-World Bank Special Programme of Research, Development and Research Training in Human Reproduction Department of Sexual and Reproductive Health and Research World Health Organization Geneva Switzerland; 2 Department of Biostatistics National Institute for Research in Reproductive and Child Health Indian Council of Medical Research Mumbai India; 3 Department of Impact Evaluation, Health System and Policy Analysis Ifakara Health Institute Dar es Salaam United Republic of Tanzania; 4 Department of Epidemiology and Community Health, College of Health Sciences University of Ilorin and University of Ilorin Teaching Hospital Ilorin Nigeria; 5 Department of Sexual and Reproductive Health and Research World Health Organization Geneva Switzerland; 6 Institute of Child Health University of Ibadan Ibadan Nigeria

**Keywords:** contraception, family planning, stock-out, COVID-19, facility assessment

## Abstract

**Background:**

The COVID-19 pandemic and the associated social restrictions may have disrupted the provision of essential services, including family planning (FP) and contraceptive services. This protocol is adapted from a generic study protocol titled “Health systems analysis and evaluations of the barriers to availability and readiness of sexual and reproductive health services in COVID-19 affected areas,” conducted by the World Health Organization (WHO) Department of Reproductive Health and Research.

**Objective:**

This study aims to assess the availability and use of FP and contraceptive services in primary health facilities during and after the COVID-19 pandemic; assess the risk perceptions of COVID-19 stigma, barriers to access, and quality of services from clients’ and providers’ perspectives in the COVID-19–affected areas; and assess the postpandemic recovery of the facilities in the provision of FP and contraceptive services.

**Methods:**

In-depth interviews will be conducted with clients—women in the reproductive age group and their male partners who visit the selected health facilities for FP and contraceptive services—and health providers (the most knowledgeable person on FP and contraceptive service provision) at the selected health facilities. Focus group discussions will be conducted with clients at the selected health facilities and in the community. The in-depth interviews and focus group discussions will help to understand clients’ and health service providers’ perspectives of FP and contraceptive service availability and readiness in COVID-19–affected areas. A cross-sectional health facility assessment will be conducted in all the selected health facilities to determine the health facility infrastructure’s ability and readiness to provide FP and contraceptive services and to capture the trends in FP and contraceptive services available during the COVID-19 pandemic. Scientific approval for this study is obtained from the WHO Research Project Review Panel, and the WHO Ethics Review Committee has given ethical approval in the 3 countries.

**Results:**

Using a standardized research protocol will ensure that the results from this study can be compared across regions and countries. The study was funded in March 2021. It received ethics approval from the WHO Ethics Review Committee in February 2022. We completed data collection in September 2022. We plan to complete the data analysis by March 2023. We plan to publish the study results by Summer 2023.

**Conclusions:**

The findings from this study will provide a better understanding of the impact of the COVID-19 pandemic on FP and contraceptive services at the facility level, which will help policy makers and health managers develop and strengthen FP policies and services in health facilities to be more responsive to community needs.

**International Registered Report Identifier (IRRID):**

DERR1-10.2196/43329

## Introduction

### Background

COVID-19 was first reported in December 2019 and has since spread rapidly around the globe, being declared a pandemic by the World Health Organization (WHO) on 11 March 2020 [1]. The pandemic led to an unprecedented increase in demand on health systems to care for people infected with COVID-19.

Lessons from the Ebola and Zika virus outbreaks have highlighted the severe disruptions in sexual and reproductive health (SRH) services that expose women and girls in particular to preventable health risks [2]. The United Nations Population Division has suggested that the COVID-19 pandemic can result in 60 million fewer users of contraceptive methods worldwide, with projected declines expected to be most significant for injectables (–20%), condoms (–10%) and the pill (–10%) [3]. It is expected that during the pandemic, access to family planning (FP) services will disproportionately affect the rural populations, as supply lines and services are more thinly distributed and contraceptive methods that require medical consultations will have particularly noticeable falls, whereas methods that can be used independently will be less affected [3,4]. According to the United Nations Population Fund, overwhelmed with COVID-19 cases, clinical staff may not have the time or personal protective equipment needed to provide FP counselling and commodities [5]. Women refrain from visiting health facilities due to movement restrictions or fears about COVID-19 exposure. Meanwhile, supply chain disruptions are limiting the availability of contraceptives in many places [6]. It is anticipated that significant levels of lockdown-related disruption over 6 months could leave 47 million women in low- and middle-income countries unable to use modern contraceptives, leading to a projected 7 million additional unintended pregnancies [7]. Furthermore, studies have modelled the potential impacts, showing that even a 10% reduction in essential SRH services could lead to an estimated 15 million unintended pregnancies, 3.3 million unsafe abortions, and 29,000 additional maternal deaths during the next 12 months [7-9].

There is some scientific evidence on the impact of COVID-19 on FP and contraceptive services. A recently published scoping review [10] included 55 studies on the impact of COVID-19 on contraceptive services in low- and middle-income countries. Nearly all studies reported a decline in contraceptive service provision of varying magnitudes, but severe disruptions were relatively uncommon or of limited duration. Most included studies showed declines in at least some indicators of access to contraceptive services during the COVID-19 pandemic. Another scoping review on the global impact of the COVID-19 pandemic on SRH services included 24 studies that showed a decrease in access or use of contraceptive services, especially long-acting reversible contraception, evenly distributed across various contexts and populations [11]. A systematic review reported substantial decreases in contraceptive access and use in all 5 included studies on the impact of the COVID-19 pandemic and the associated lockdowns [12].

This research protocol details a mixed methods study to evaluate the impact of the COVID-19 pandemic on the primary health system’s capacity to provide FP and contraceptive services and the perceptions of clients and health care providers about the access and use of FP and contraceptive services in 3 countries: India, Nigeria, and Tanzania. This protocol has been adapted from a generic study titled “Health systems analysis and evaluations of the barriers to availability and readiness of sexual and reproductive health services in COVID-19 affected areas” conducted by the WHO Department of Reproductive Health and Research [13].

### Study Aims and Objectives

The study aims to assess the availability of FP and contraceptive services in primary health facilities during the COVID-19 pandemic. The specific study objectives are the following:

- To explore the availability of FP and health facilities’ readiness to provide FP and contraceptive services in regions most affected by the COVID-19 pandemic.
- To assess the risk perceptions of COVID-19 stigma as well as barriers to access, use, and quality of services from clients’ and providers’ perspectives in the COVID-19–affected areas.
- To assess the postpandemic recovery of the facilities in the provision of FP and contraceptive services.

## Methods

### Study Design

This is a mixed methods study that uses both quantitative and qualitative methods. In-depth interviews (IDIs) and focus group discussions (FGDs) will be used to understand clients’ and health service providers’ perspectives of FP and contraceptive service availability and readiness in COVID-19–affected areas. A cross-sectional health facility assessment will be conducted to determine the health facility infrastructure’s ability and readiness to provide FP and contraceptive services and to capture the trends in FP and contraceptive services availability during the COVID-19 pandemic. Table 1 summarizes the study design, analysis plan, and the expected outcomes.

**Table 1:** Overview of study methodology.

Study type and method		Respondent	Study setting	Study tools	Analysis plan	Variable measured
**Qualitative study**						
	In-depth interview	Women of reproductive age Men of reproductive age	Health facility	Semistructured interview guide for women Semistructured Interview guide for men	Computer- assisted thematic analysis using NVivo or ATLAS.ti	Knowledge of COVID-19 Gaps in service provision Risk perception, beliefs, and concerns Enablers and barriers in availing services
	In-depth interview	Health care providers	Health facility	Semistructured interview guide for health care providers	Computer- assisted thematic analysis using NVivo or ATLAS.ti	Knowledge of COVID-19 Gaps in service provision Risk perception, beliefs, and concerns Recovery in service availability
	Focus group discussion	Women in reproductive age Men in reproductive age	Health facility	Focus group discussion topic guide for women Focus group discussion topic guide for men	Computer- assisted thematic analysis using NVivo or ATLAS.ti	Knowledge of COVID-19 Gaps in service provision Risk perception, beliefs, and concerns Enablers and barriers in availing services
	Focus group discussion	Women in reproductive age	Community	Focus group discussion topic guide for women	Computer- assisted thematic analysis using NVivo or ATLAS.ti	Knowledge of COVID-19 Gaps in service provision Risk perception, beliefs, and concerns Enablers and barriers in availing services
**Quantitative study**						
	Health facility assessment	Senior Health care provider or administrator	Health facility	Health facility assessment questionnaire	Database will be developed. SPSS will be used for descriptive analysis.	Knowledge of COVID-19 Gaps in service provision Risk perception, beliefs, and concerns Recovery in service availability
	General client information	All men and women who participate in Focus group discussions and in-depth interviews	Health facility	General client questionnaire	Database will be developed. SPSS will be used for descriptive analysis.	Sociodemographic information

### Study Setting

This study will be conducted in India, Nigeria, and Tanzania by the Indian Council of Medical Research—National Institute of Research in Reproductive and Child Health, the University of Ilorin Teaching hospital, and the Ifakara Health Institute, respectively. These 3 countries are selected as they are a part of the WHO FP Accelerator project [14], and we leveraged the support from the project funds to support the research. Other criteria included the willingness of the Ministry of Health to participate in the study as well as the capability and capacity of research institutions in the 3 countries.

In each country, the research team will select study sites based on geographical location (ease of access and areas with a high incidence of COVID-19 cases), organization of FP and contraceptive services, and epidemic status, where the COVID-19 pandemic is likely to have significantly affected service delivery.

### Study Population

The study population will be women and their male partners who visit the selected primary health facilities for FP and contraceptive services during the study period. It also includes health providers who provide FP and contraceptive services at the selected health facilities. In countries where it is not customary for men to accompany their partners when they seek FP and contraceptive services, other options will be used to recruit male partners.

### Health Facility Selection

The minimum criteria should be the selection of primary health care facilities providing FP services and the availability of human resources qualified to provide FP and contraceptive services. Rural and urban representation of the primary health facilities in each region will be ensured.

### Qualitative Study

#### Selection of Women and Male Clients

Women and their male partners seeking FP and contraceptive services at the selected facilities will be approached to participate in the IDIs and FGDs. The women and men will be purposively selected, if they fulfil the following criteria: (1) being of reproductive age (18-49 years), (2) having sought or received FP and contraceptive services from the selected primary health facilities, and (3) being comfortable discussing their experience seeking FP and contraceptive services in a group (for FGDs).

A health provider from the selected health facility, who is not a part of the study, will help identify potential participants based on the inclusion criteria and ascertain their willingness to participate in the study. Participants will be purposively selected to obtain a sample of women and men from all socioeconomic backgrounds in the area. The health provider will identify potential participants from among the women visiting the facility for FP and contraceptive services and will ask the women their age and willingness to participate in an FGD using screening questions to check for eligibility. The provider will approach the clients after they have been attended to or while waiting to be attended. The screening will occur in a private room selected by the provider and agreed upon by the study team. Clients who agree to participate will be introduced to the study research team. All participants will be given comprehensive information about the study, and all their questions and concerns will be addressed. Written informed consent will be obtained from all participants.

To provide a holistic picture of the knowledge and perceptions about FP and contraceptive services during the COVID-19 pandemic in the community, FGDs will be conducted with women in the community who have not obtained FP and contraceptive services from the selected public health facility but may have sought FP services from other private or public facilities. The community health workers will help identify participants for this FGD. Interested women will be asked to contact the study team.

The study will recruit a purposive sample of 10 to 15 women (or until saturation) and 6 to 12 male partners (or until saturation) per health facility for the IDIs. At each health facility, one FGD will be conducted with women clients, one with male partners, and one with women in the community.

#### Selection of Health Care Providers

Health care providers will be purposively selected to participate in the IDIs. Health care providers need to fulfil the following inclusion criteria: (1) those who deliver FP and contraceptive services, (2) are knowledgeable about the readiness and availability of FP and contraceptive services, and (3) are stationed in the FP clinic and have been working at the clinic for at least six months before the pandemic started. The health care provider will be selected after obtaining information from the facility in charge. Participation will be voluntary, and only the health care providers who accept to participate after talking with the study gatekeepers will be referred to the study team for interviews. Approximately 1 to 2 health care providers per health facility will be selected to participate in the IDIs. Depending on the norms and standards for providing FP services in different country contexts, they will include medical doctors, nurses, midwives, nurse assistants, allied health professionals, and other cadres.

### Quantitative Study

A general questionnaire for clients will be used to collect sociodemographic data from all participants of the qualitative study. The most knowledgeable person on FP and contraceptive service provision in each selected health facility (senior health care provider or administrator) will be selected to assist in completing the health facility assessment.

### Data Collection

#### Qualitative Study

Qualified researchers with experience in conducting IDIs and FGDs will be selected and trained to ensure the validity of data collection, build trust with participants, and protect participants’ privacy and confidentiality. Male facilitators will facilitate the IDIs and FGDs with men, and female facilitators will facilitate FGDs and IDIs with women. The interviews will be conducted in a place that is convenient and safe for the participants to discuss the subject matter freely. The interviews will commence after the introduction and informed consent processes are completed. To ensure all information is captured, interviews will be audio-recorded. The researcher will also take notes on the participant’s nonverbal communication during the interview. We expect each IDI to last approximately 40-60 minutes and each FGD to last 60-90 minutes.

To guarantee the anonymity of the participants, any personal or family identifiers will be removed. After each IDI or FGD, the moderators will send the recording to the team supervisor, who will store them securely for transcription. To protect the data from unintended use, the audio files will be encrypted and sent to an electronic database, which will also be shared with the WHO. Therefore, only the research team will be authorized to listen to the recordings. These audio recordings will be retained until the researcher has transcribed and checked them for accuracy, after which they will be destroyed.

#### Quantitative Study

The health facility assessment survey will be implemented using a cross-sectional survey design. All facilities identified in the selection phase will be assessed. The assessment will include a review of health facility records of FP and contraception to assess the availability, type, and range of commodities offered prior to, during, and after the pandemic. Records for 6 months prior to the start of the pandemic will be reviewed to determine the recovery in service availability and readiness in health facilities. The assessment will also highlight gaps or service delivery issues during the COVID-19 pandemic. The quantitative data will be collected on paper in India and electronically in Nigeria and Tanzania.

Pilot studies will be conducted in all 3 study sites to ensure the validity of the questionnaires in the local context.

#### Data Collection Tools

The qualitative study includes 5 data collection tools: (1) IDI topic guide for women, (2) IDI topic guide for men, (3) IDI topic guide for health care providers, (4) FGD topic guide for women, and (5) FGD topic guide for men. These tools will help understand the psychosocial effects of COVID-19 on fertility desires, knowledge of COVID-19, risk perceptions and concerns about the COVID-19 effects on SRH, care-seeking behaviors, experiences and barriers in accessing FP and contraceptive services, the influence of men on access to FP services, and the role the male partners play when their partners require contraception.

The quantitative study includes 2 data collection tools: (1) a general questionnaire to collect sociodemographic information from all participants of the qualitative study and (2) a health facility assessment questionnaire. The health facility assessment questionnaire follows and adopts the WHO’s Service Availability and Readiness Assessment tool [15] and the health facility checklist developed by the WHO Health Emergency department [16] for assessment of infrastructure and services. This will help to understand how health systems delivered FP and contraceptive services during the emergency response to the COVID-19 pandemic. The following indicators will be used to assess the availability and readiness of health facilities in the provision of FP and contraceptive services: (1) policies and plans, (2) services maintenance and referrals, (3) infrastructure, (4) commodities, and (5) human resources.

The data collection tools will be translated into local languages: Yoruba and Hausa in Nigeria, Kiswahili in Tanzania, and Hindi and Marathi in India.

### Data Analysis

The audio-recorded data will be transcribed verbatim and deidentified using ID numbers. Interviews conducted in local languages will be translated to English and back-translated to ensure there is no distortion of information. The data will be analyzed using general content analysis approach according to the suggested steps by Elo and Kyngäs [17]. This will involve reading through the verbatim transcripts and notes to understand what was being expressed by the participants, noting down emerging themes and patterns in the data, and condensing the data into codes. The codes will be used to generate categories and condensation of related categories into themes that convey the meaning of the data. The coding method and progress will be systematically discussed between the researchers and the project coordinator. The analysis will be done using NVivo software (version 12; QSR International). Two researchers will analyze the same data set and be guided by general questions, specific questions, and comparisons. The analysis will begin while data collection is ongoing to assess progress and determine any data collection problems and patterns.

Quantitative data from the facility assessment survey will be entered into an electronic database using SPSS (IBM Corp). Descriptive analysis will be used to illustrate the basic characteristics of the different facilities, including the monthly number of clients, types of procedures provided, number of medical staff, and stocks of drugs. In Tanzania, the Open Data Kit platform will be used to collect data digitally on tablets, and the KoboCollect App will be used in Nigeria on the research staff’s phones for data collection.

### Ethics Approval

Scientific approval for this study in 3 countries has been obtained from the WHO Research Project Review Panel. The WHO Ethics Review Committee has given ethical approval for this study in 3 countries (protocol IDs CERC.0103K, CERC.0103J, and CERC.0103I). Each study site is required to obtain ethical approvals based on the specific requirements in each country.

## Results

We expect to gain more insight into the impact of the COVID-19 pandemic on FP and contraceptive services at the primary health care level and the trends in contraceptive uptake during and after the pandemic. The study was funded in March 2021 and approved by the WHO Ethics Review Committee in February 2022. Data collection was completed between April and September 2022. The project is ongoing. Expected results will be published in summer 2023.

## Discussion

### Expected Outcomes

This paper outlines the design of a multicountry study determining the impact of the COVID-19 pandemic on FP and contraceptive services at the primary health care level in India, Nigeria, and Tanzania. This study will explore the availability of FP and health facility readiness to provide FP and contraceptive services; it will assess the risk perceptions of COVID-19 stigma and barriers to access and use services as well as their quality from clients’ and providers’ perspectives; it will also assess the postpandemic recovery of the facilities in the provision of FP and contraceptive services.

A cross-sectional study was conducted across 10 states of Nigeria to determine the impact of the COVID-19 pandemic on the provision of SRH services at primary health facilities [18]. Findings showed that 97.7% of the 307 primary health centers offered FP services before the pandemic and lockdown, which decreased to 95.8% during the lockdown and reduced to 92.5% after the lockdown. A rapid qualitative study among young men and couples in 2 districts from 2 states of India (Bihar and Uttar Pradesh) showed a marked increase in the demand and use of condoms compared to pre–COVID-19 times, while women avoided the use of oral contraceptive pills due to the misperception of its ill effects on their health [19]. A mixed methods study in 3 purposively selected districts from 3 regions of Tanzania (Unguja, Pemba, and Dar es Salaam) revealed low uptake of FP services because of changes in clinic schedules, fear of COVID-19 infection, and unavailability of contraceptives [20]. Additionally, the quantitative analysis of FP use between January and March in 2019, 2020, and 2021 showed a decrease in FP use in 2020 for continuing clients, whereas an increased trend was noted for new clients in 2020 and 2021. A web-based survey among 51 clinicians and stakeholders in 29 countries showed that 86% perceived access to contraceptive services was less or much less because of the pandemic [21]. On the other hand, a population-based survey in 4 sub-Saharan African countries (Burkina Faso, Democratic Republic of the Congo, Nigeria, and Kenya) showed that among the 7245 women surveyed, there were no deleterious effects of the pandemic on contraceptive access and use [22].

In the scoping review by Polis et al [10], the study design of most of the included studies (69%) were cross-sectional or longitudinal surveys, and many were conducted on the web or by phone due to the pandemic [10]. However, our study uses a mix of quantitative and qualitative methods to assess the health facilities’ availability and readiness to provide these services and clients’ and providers’ perceptions of the availability and readiness of these services in COVID-19–affected areas. Data in our study will be collected in person.

Polis et al [10] report the reasons for the change in the contraceptive demand as lack of awareness that contraceptives were provided during the pandemic, preferences for methods accessible with no or fewer visits to health facilities, use of condoms to attempt prevention of COVID-19 transmission, and the stigma that respondents perceived or experienced at health facilities. The reasons for disruptions in the provision of contraceptives were reported as reduced availability or stock-outs, not being able to offer provider-administered methods, health providers advising FP clients not to seek services at health facilities, fewer clients permitted in clinics due to social distancing measures, and service disruptions across multiple humanitarian contexts due to mandatory restrictions, lockdowns, and curfews. Key barriers to accessing contraceptive services during the pandemic included fear of infection in general and in health facilities, lack of transport or travel-related restrictions, costs, increased waiting times, limited home visits by health workers who would normally provide contraceptive services, lack of supplies, and for adolescents in humanitarian settings, the compounding effect of other pandemic challenges, such as being out of school and isolated from peers [10].

### Limitations

We anticipate certain limitations for this study. The study focuses on primary health care facilities and only in a few sites per country. The information gained might not be generalizable to the situation in other public facilities and private facilities as well as all over the country. However, information on FP access at this level is necessary because it indicates the situation the most vulnerable in the society are facing and would call for prompt intervention. The use of a standardized research protocol can ensure that the results from these studies can be compared across regions and countries and will potentially improve the quality of observational studies by identifying and minimizing biases. Since most of the countries are out of the pandemic, there can be recall bias because participants would be reproducing the experiences from the past 2 years. However, as the pandemic and lockdowns are rare events, many might remember their personal experiences.

### Conclusions

The findings from this study will provide a better understanding of the impact of the COVID-19 pandemic on FP and contraceptive services at the subnational and facility level, which will help policy makers and health managers develop and strengthen policies and services to be more responsive to community needs. These policies and services will ensure the availability of a contraceptive method mix that is accessible, affordable, and women-centered during emergency situations like the COVID-19 pandemic.

## Abbreviations

FGD: focus group discussion
FP: family planning
IDI: in-depth interview
SRH: sexual and reproductive health
WHO: World Health Organization
